# Decision-making in the multiphase optimization strategy: Applying decision analysis for intervention value efficiency to optimize an information leaflet to promote key antecedents of medication adherence

**DOI:** 10.1093/tbm/ibae029

**Published:** 2024-05-25

**Authors:** Sophie M C Green, Samuel G Smith, Linda M Collins, Jillian C Strayhorn

**Affiliations:** Behavioural Oncology Research Group, Leeds Institute of Health Sciences, University of Leeds, Leeds, UK; Behavioural Oncology Research Group, Leeds Institute of Health Sciences, University of Leeds, Leeds, UK; Department of Social and Behavioral Sciences, New York University School of Global Public Health, New York, NY, USA; Department of Social and Behavioral Sciences, New York University School of Global Public Health, New York, NY, USA

**Keywords:** intervention optimization, multiphase optimization strategy, decision-making, factorial optimization trial, Bayesian decision analytics, breast cancer

## Abstract

Advances in the multiphase optimization strategy (MOST) have suggested a new approach, decision analysis for intervention value efficiency (DAIVE), for selecting an optimized intervention based on the results of a factorial optimization trial. The new approach opens possibilities to select optimized interventions based on multiple valued outcomes. We applied DAIVE to identify an optimized information leaflet intended to support eventual adherence to adjuvant endocrine therapy for women with breast cancer. We used empirical performance data for five candidate leaflet components on three hypothesized antecedents of adherence: beliefs about the medication, objective knowledge about AET, and satisfaction with medication information. Using data from a 2^5^ factorial trial (*n* = 1603), we applied the following steps: (i) We used Bayesian factorial analysis of variance to estimate main and interaction effects for the five factors on the three outcomes. (ii) We used posterior distributions for main and interaction effects to estimate expected outcomes for each leaflet version (32 total). (iii) We scaled and combined outcomes using a linear value function with predetermined weights indicating the relative importance of outcomes. (iv) We identified the leaflet that maximized the value function as the optimized leaflet, and we systematically varied outcome weights to explore robustness. The optimized leaflet included two candidate components, side-effects, and patient input, set to their higher levels. Selection was generally robust to weight variations consistent with the initial preferences for three outcomes. DAIVE enables selection of optimized interventions with the best-expected performance on multiple outcomes.

## Teaser

The recently proposed decision-making framework for use in intervention optimization, DAIVE, was applied to select a new optimized information leaflet to support eventual medication adherence in women with breast cancer.

**Implications**

**Practice:** When interventions are successfully optimized for effectiveness on the multiple different outcomes that matter to investigators, patients, practitioners, and/or other interested parties, such interventions are better equipped to accomplish the desired public health impacts.

**Policy:** DAIVE is applicable to a wide range of optimization scenarios, supporting the larger mission of identifying efficient interventions that can be expected to produce the desired outcomes with minimal waste.

**Research:** Investigators using data from a factorial trial to select an optimized intervention can use DAIVE to base decision-making on more than one valued outcome.

## Introduction

Multicomponent interventions are used to promote a wide range of health behaviors [1–4]. For example, in secondary cancer prevention, complex interventions may aim to support medication adherence, with individual intervention components targeting different barriers to adherence [5]. The intervention components that make up a complex intervention incur costs, such as money, time, participant burden, or cognitive load. Removing ineffective or underperforming components improves intervention efficiency, saving resources that could be allocated elsewhere [6].

The multiphase optimization strategy (MOST) is an engineering-inspired framework for optimizing complex interventions such that they are not only effective but also readily implementable, e.g. because they are efficient [6, 7]. In MOST, intervention components are considered candidates for inclusion in an eventual intervention package. The individual and combined effects of the candidate components are estimated in an optimization randomized control trial (ORCT), often using a design from the factorial family of experiments [8]. Based on the empirical results of the ORCT, an optimized intervention—i.e. a combination of the candidate components that is, ideally, effective, and efficient, or that otherwise accomplishes the objective for optimization—is selected [7, 9]. A key purpose of the ORCT is to provide information useful in identifying and removing ineffective or underperforming candidate components.

A component screening approach (CSA) was initially recommended as a way to select an optimized intervention based on empirical ORCT data [9, 10]. However, CSA has limitations [11]. First, CSA relies on systematic interpretation of certain important main and interaction effects estimated in the ORCT using a process that is difficult to apply when there is more than one outcome variable of interest. This is an important limitation, as multicomponent interventions frequently have objectives that involve multiple valued outcomes [11]. For example, in the context of medication adherence, outcomes such as quality of life and physical functioning may also be important, in addition to adherence [12]. Second, the systematic screening process used in CSA, detailed and demonstrated in Green *et al*. [13], relies on arbitrary thresholds (usually, but not always, statistical significance thresholds) to determine which estimated main and interaction effects are “important” enough to contribute to decision-making. This can make the approach prone to missing relevant information from near-threshold effects. Finally, the systematic interpretation of main and interaction effects in CSA frequently requires difficult judgment calls. This can occur when two different important effects indicate contradictory decisions about components (e.g. one indicating a component should be included and another indicating a component should not be included). CSA does not dictate how contradictory screening decisions should be dealt with.

Recent methodological advances in MOST have suggested an alternative approach for selecting optimized interventions [11, 14]. Using the concept of posterior expected value from the Bayesian decision sciences [15], this alternative approach, decision analysis for intervention value efficiency (DAIVE), overcomes limitations of CSA. DAIVE can readily incorporate multiple outcome variables; outcomes are combined using a value function that reflects the decision-maker’s preferences (e.g. giving some outcomes more importance than others, as appropriate) [11]. As explained more in the Methods section, DAIVE does not rely on arbitrary significance cutoffs, and therefore makes use of all available empirical information. Moreover, DAIVE does not rely so heavily on interpretation of individual effects, as expected outcomes can be estimated without preliminary screening of components. An optimized intervention can be chosen that maximizes expected value, or that strategically balances expected values with other relevant criteria (e.g. resource constraints).

Green *et al*. [13] previously applied CSA to optimize an information leaflet intended to support women with early-stage breast cancer who are prescribed medication called adjuvant endocrine therapy (AET). AET can reduce breast cancer recurrence by up to 30%, and mortality by up to 40% compared with no endocrine therapy [13, 16, 17]. However, maintaining adherence to AET can be challenging, e.g. due to side-effects such as hot flushes and joint pains, which are commonly reported in women taking AET [18]. The information leaflet components were designed to target key barriers to AET adherence [19–24], e.g. the presence of concerns about AET or low perceived necessity AET. A 2^5^ factorial trial estimated the individual and combined effects of factors representing five candidate leaflet components: (i) diagrams about how AET works; (ii) visual representations of the benefits of AET; (iii) detailed information about the prevalence of AET side-effects; (iv) answers to common concerns about AET; and (v) quotes and pictures from breast cancer survivors [13]. Effects were estimated on three outcomes: (i) women’s *medication beliefs* about AET, reflecting the balance between concerns and necessity beliefs [25, 26]; (ii) their *objective knowledge* about AET; and (iii) their *satisfaction with information* received about AET. Of these three outcomes, *medication beliefs* was considered primary, given well-established relationships between beliefs and adherence [21, 25, 27]. *Objective knowledge* and *satisfaction with information* are also hypothesized antecedents of eventual adherence to AET, but these relationships are less consistently reported. Lower knowledge about AET has been associated with lower adherence [28], and women prescribed AET frequently report receiving insufficient information about the medication and its side-effects [22, 23, 29, 30].

When Green *et al*. [11] initially identified an optimized leaflet (i.e. just before the latest advances in optimization decision-making were published), CSA required the use of a single outcome variable for selection of an optimized intervention. As a result, Green *et al*. [11] identified a leaflet that was optimized for effects on only the primary outcome, *medication beliefs*. With this approach, components with strong effects on, say, *knowledge* but not *medication beliefs* would not be identified. To base decisions about component performance on only one of three-valued outcomes (i.e. *medication beliefs* only) could mean overlooking potentially useful candidate components.

The purpose of the present study was to apply DAIVE to optimize an information leaflet for effectiveness on multiple valued hypothesized antecedents of adherence to AET. In a secondary analysis of the 2^5^ factorial trial [11], we incorporated empirical information about component performance on all three outcome variables in the 2^5^ factorial trial [13]: *medication beliefs*, *objective knowledge*, and *satisfaction with information*. As described in more detail below, we gave the primary outcome, *medication beliefs*, greater importance relative to the other two outcomes, and we further determined that *objective knowledge* was relatively more important than *satisfaction with information*. To examine the robustness of the decision made, we systematically varied the outcome weights used to assign differential importance to the three outcomes, and observed whether this changed the decision about identification of the optimized leaflet.

## Methods

### Experimental design

The trial used a full 2^5^ factorial design (2 × 2 × 2 × 2 × 2) to estimate the individual and combined effects of five candidate intervention components [13]. Each candidate component was operationalized as a two-level factor: (i) *diagrams* detailing the mechanisms of AET (factor levels: on/off); (ii) *benefits*, with visual icon arrays detailing the benefits of AET (factor levels: enhanced/basic); (iii) *side-effects*, with detailed information about the prevalence of AET side-effects (factor levels: enhanced/basic); (iv) *concerns*, with answers to common concerns about AET (factor levels: on/off); and (v) *patient*, with quotes and pictures from breast cancer survivors (factor levels: on/off) (full descriptions available in Supplementary Appendix 1). Factor levels for benefits and side-effects were enhanced versus basic, as we felt any optimized version of the leaflet should contain at least a basic level of information about the benefits and side-effects of AET. A sample of *n* = 1603 healthy women were randomized to 32 experimental conditions, each comprising a unique combination of factor levels (Table 1). Participants were required to be over 18 and able to read English, and did not require a diagnosis of breast cancer to participate. Full details about the candidate components, factors, sample recruitment, and demographics are available elsewhere [13]. Study materials are available online (DOI 10.17605/OSF.IO/AG7YK).

**Table 1:** Experimental conditions in 2^5^ factorial design and number randomized to each condition

	Constant component	*Diagrams*	*Benefits*	*Side-effects*	*Concerns*	*Patient*	Number randomized
1	Yes	Yes	Enhanced	Enhanced	Yes	Yes	55
2	Yes	Yes	Enhanced	Enhanced	Yes	No	54
3	Yes	Yes	Enhanced	Enhanced	No	Yes	53
4	Yes	Yes	Enhanced	Enhanced	No	No	38
5	Yes	Yes	Enhanced	Basic	Yes	Yes	53
6	Yes	Yes	Enhanced	Basic	Yes	No	56
7	Yes	Yes	Enhanced	Basic	No	Yes	47
8	Yes	Yes	Enhanced	Basic	No	No	58
9	Yes	Yes	Basic	Enhanced	Yes	Yes	45
10	Yes	Yes	Basic	Enhanced	Yes	No	57
11	Yes	Yes	Basic	Enhanced	No	Yes	42
12	Yes	Yes	Basic	Enhanced	No	No	50
13	Yes	Yes	Basic	Basic	Yes	Yes	54
14	Yes	Yes	Basic	Basic	Yes	No	41
15	Yes	Yes	Basic	Basic	No	Yes	49
16	Yes	Yes	Basic	Basic	No	No	63
17	Yes	No	Enhanced	Enhanced	Yes	Yes	45
18	Yes	No	Enhanced	Enhanced	Yes	No	55
19	Yes	No	Enhanced	Enhanced	No	Yes	56
20	Yes	No	Enhanced	Enhanced	No	No	42
21	Yes	No	Enhanced	Basic	Yes	Yes	61
22	Yes	No	Enhanced	Basic	Yes	No	52
23	Yes	No	Enhanced	Basic	No	Yes	54
24	Yes	No	Enhanced	Basic	No	No	58
25	Yes	No	Basic	Enhanced	Yes	Yes	44
26	Yes	No	Basic	Enhanced	Yes	No	51
27	Yes	No	Basic	Enhanced	No	Yes	40
28	Yes	No	Basic	Enhanced	No	No	50
29	Yes	No	Basic	Basic	Yes	Yes	46
30	Yes	No	Basic	Basic	Yes	No	39
31	Yes	No	Basic	Basic	No	Yes	43
32	Yes	No	Basic	Basic	No	No	52

*Note.* Each component had two levels: on versus off, or enhanced versus basic. Table adapted from Green *et al*. [13].

### Outcome assessments

#### Medication beliefs

At two time points, before and after being shown a version of the information leaflet (containing a unique combination of candidate intervention components), participants responded to the Beliefs about Medicine Questionnaire-AET [26], with 10 items assessing specific medication beliefs on a 5-point scale ranging from “strongly disagree” to ‘strongly agree’ [26]. There were two subscales: necessity beliefs and concerns, with five items each. Cronbach’s alpha (α) scores, assessing internal consistency reliability, for the necessity subscale were 0.852 and 0.884 pre- and post-leaflet respectively, and 0.808 and 0.831 for the concerns subscale pre- and post-leaflet, respectively. For the *medication beliefs* outcomes (baseline and post-leaflet), a beliefs differential score was calculated by subtracting concern from necessity scores (range −20 to +20). The differential follows recommended scoring [31], and has more consistently predicted nonadherence than necessity beliefs or concerns subscales alone across conditions [32].

#### Objective knowledge

The *objective knowledge* outcome was assessed after participants viewed a version of the information leaflet using eight “true or false” items about AET. The eight statements, which involved the mechanisms of AET (two statements), the benefits of taking AET (two statements), the prevalence of side-effects (three statements), and the management of side-effects (one statement), were written to reflect key information in the leaflet. Four statements were true, and four statements were false.

#### Satisfaction with information

After viewing a version of the information leaflet, participants responded to a modified version of the original Satisfaction with Information about Medicines Scale [33]. Participants were asked to rate their satisfaction with the information about AET provided across 11 domains, such as how hormone therapy works and what the benefits of the medication are. Participants responded on a 5-point scale: “too much,” “about right,” “too little,” “none received,” or “none needed.” An overall satisfaction score ranging from 0 to 11 was calculated, whereby responses of “about right” or “none needed” (indicating satisfaction) were scored as 1, while all other responses (indicating dissatisfaction) were scored as 0 (Cronbach’s α = 0.875).

### Applying DAIVE

#### Overview

In contrast to CSA, DAIVE makes use of a Bayesian paradigm [11, 14]. First, factorial trial data on one or more outcome variables are analyzed using Bayesian factorial analysis of variance, which summarizes evidence about main and interaction effects using posterior distributions. These posterior distributions for main and interaction effects are then used to obtain posterior distributions for the expected outcomes ($\hat{Y}s$) associated with each version of the intervention under consideration (i.e. each of the 32 versions of the information leaflet comprising unique combinations of the factor levels). In further contrast to CSA, expected outcomes are estimated with no need for preliminary screening. When multiple outcomes matter, outcomes are combined using a value function, such as a simple linear weighted sum. For example, with *J* outcomes:


$$\begin{array}{l} V=\;w_{1}Y_{1}+\;w_{2}Y_{2}\;+\;\ldots \;+w_{J}Y_{J} \end{array}$$

where each weight *w*_*i*_ indicates the relative importance of that outcome *Y*_*i*_. Depending on the optimization objective [7], the optimized intervention may be the intervention that maximizes expected value. Alternatively, expected values may be strategically balanced with additional criteria (e.g. intervention delivery cost), when this fits the objective of optimization.

#### Identifying an optimized leaflet

We analyzed factorial data using R version 4.2.2 [34] and the brms package [35]. We fit a regression model (see code in Appendix 2) for each of the three outcomes: *medication beliefs*, *objective knowledge*, and *satisfaction with information*. Each model included all 31 main and interaction effects and was equipped with non-informative *N*(0,5) priors. To estimate main and interaction effects on the primary outcome, *medication beliefs* after viewing the leaflet, we also controlled for baseline *medication beliefs*.

Using the steps modeled by Strayhorn *et al*. [14], we scaled and assigned weights to outcomes using a swing weighting exercise [36]. First, we identified the swing in *medication beliefs* as the most important, as medication beliefs are one of the most consistently reported barriers to AET adherence [21]. Following conventions in swing weighting, this meant that we assigned *medication beliefs* 100 points. We identified the swing in *objective knowledge* as the next-most important—i.e. half as important as *medication beliefs*—given some (less consistently reported) evidence suggesting that knowledge of AET is associated with nonadherence to AET [28]. We therefore gave objective knowledge 50 points. Finally, we identified the swing in *satisfaction with information* as half as important as *objective knowledge* (1/4 as important as *medication beliefs*), given potential subjectivity in the satisfaction outcome. We therefore gave *satisfaction with information* 25 points. We calculated outcome weights by dividing the points for a given outcome (100, 50, or 25) by the total number of points for all outcomes (175). This gave us the following value function (rounded to the nearest 0.1):


$$\begin{array}{l} V=0.6Y_{BMQ\text{\textasciitilde{}}\text{-}AET}+0.3Y_{Knowledge}+0.1Y_{SIMS}. \end{array}$$

We then put all outcomes on a 0–1 scale, where 0 was worst and 1 was best.

We applied the value function to estimate expected values for each alternative intervention (i.e. each information leaflet, containing a unique combination of factor levels), and we identified the leaflet with the largest expected value as the optimized information leaflet. In other words, we chose to advance intervention efficiency by using the estimated expected values to identify components that were—or were not—worth including in the optimized leaflet, with the goal of removing components that were redundant (or worse, harmful) in contributing to expected value.

#### Exploring robustness

By systematically varying the points assigned to outcomes in the swing weighting exercise, we repeated the identification of an optimized leaflet using value functions reflecting different preferences about the three outcomes. In general, we wanted to uphold our initial determinations about the relative importance of outcomes (i.e. that all three outcomes are important, and *medication beliefs* > *objective knowledge* > *satisfaction with information*), particularly regarding *medication beliefs* as the primary outcome. Our main goal in exploring the robustness of the choice of optimized intervention was to examine the extent to which the decision might change as a function of variation in the weights assigned to the additional two outcomes, *objective knowledge* and *satisfaction with information*. Therefore, we approached this systematic variation using the following steps:

*Step 1: Systematically reducing the importance of secondary outcomes*

Beginning with our initial preferences (*medication beliefs*: 100 points; *objective knowledge*: 50 points; *satisfaction with information*: 25 points) as a starting point, we reduced the importance of *objective knowledge* and *satisfaction with information*, relative to *medication beliefs*. We did this by incrementally reducing the points for *satisfaction with information* (i.e. the less important of the secondary outcomes) first, holding all other points constant, until *satisfaction with information* had very small importance (one-twentieth the importance of *medication beliefs*). We then incrementally reduced the points for *objective knowledge*, again holding all other points constant, until objective knowledge also had very small importance (one-twentieth the importance of *medication beliefs*). We made the reductions in this order to preserve the ranking of the importance of the three outcomes. Across the incremental changes, we observed whether the selected optimized intervention changed as the importance of the secondary outcomes decreased (eventually, to the point that the secondary outcomes were both minimally important).

*Step 2: Systematically increasing the importance of the secondary outcomes*

Beginning again with our initial preferences (*medication beliefs*: 100 points; *objective knowledge*: 50 points; *satisfaction with information*: 25 points) as the starting point, we increased the importance of *objective knowledge* and *satisfaction with information*, relative to *medication beliefs*. This time we incrementally increased the points for *objective knowledge* (i.e. the more important of the secondary outcomes) first, holding all other points constant, until *objective knowledge* had importance equal to the primary outcome, *medication beliefs*. We then incrementally increased the points for *satisfaction with information*, holding the other points constant, until all three outcomes had equal importance. Again, we observed whether the selected optimized intervention changed as the importance of the secondary outcomes increased (eventually, to the point that the three outcomes were equally important).

Finally, to contextualize these systematic variations, we also considered select preference scenarios that differed markedly from our initial preferences for the three empirical outcomes, such as one scenario in which the relative importance of the secondary outcomes were reversed.

## Results

**Figure 1:**
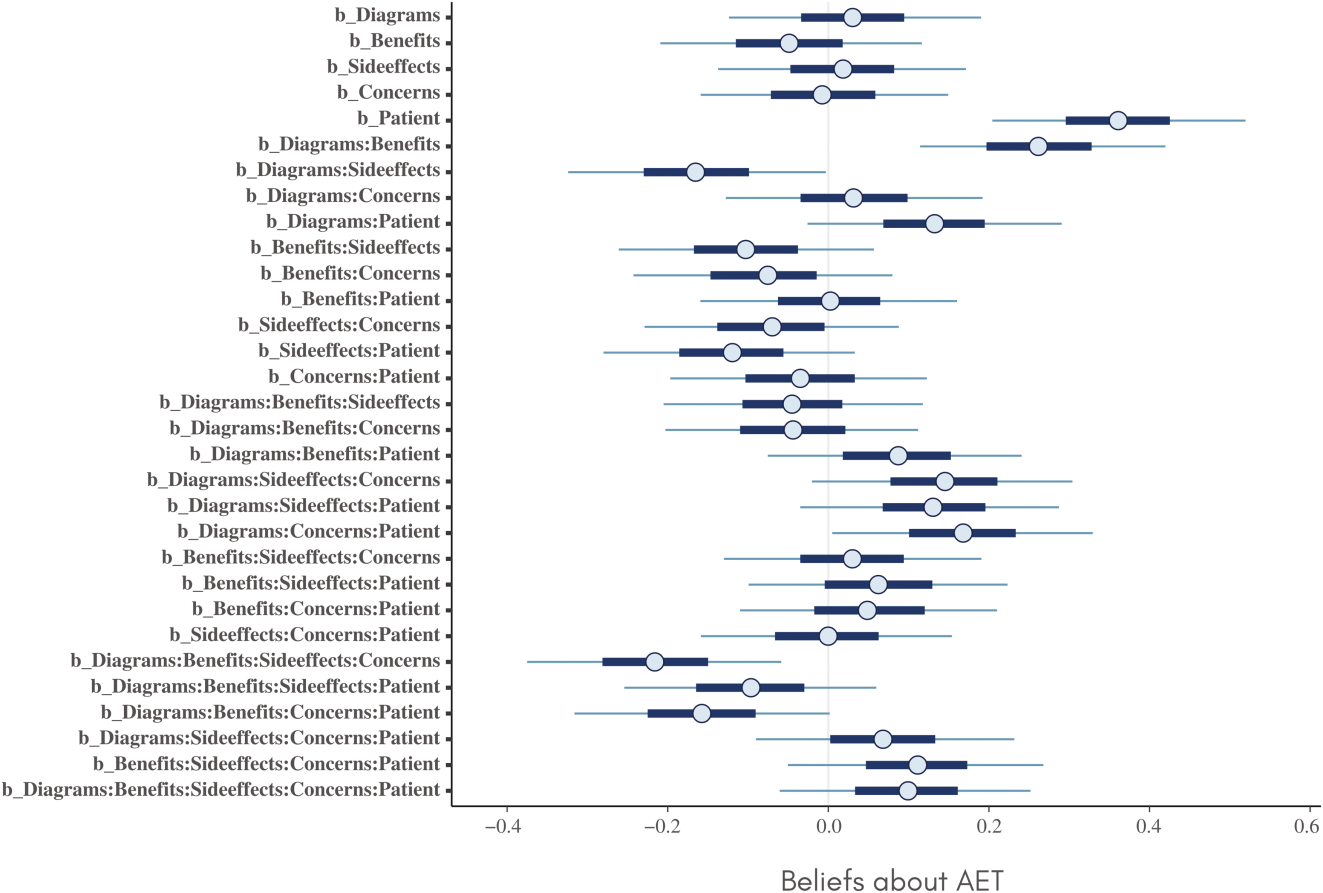
Credible intervals for the estimated main and interaction effects on beliefs about AET.

**Figure 2:**
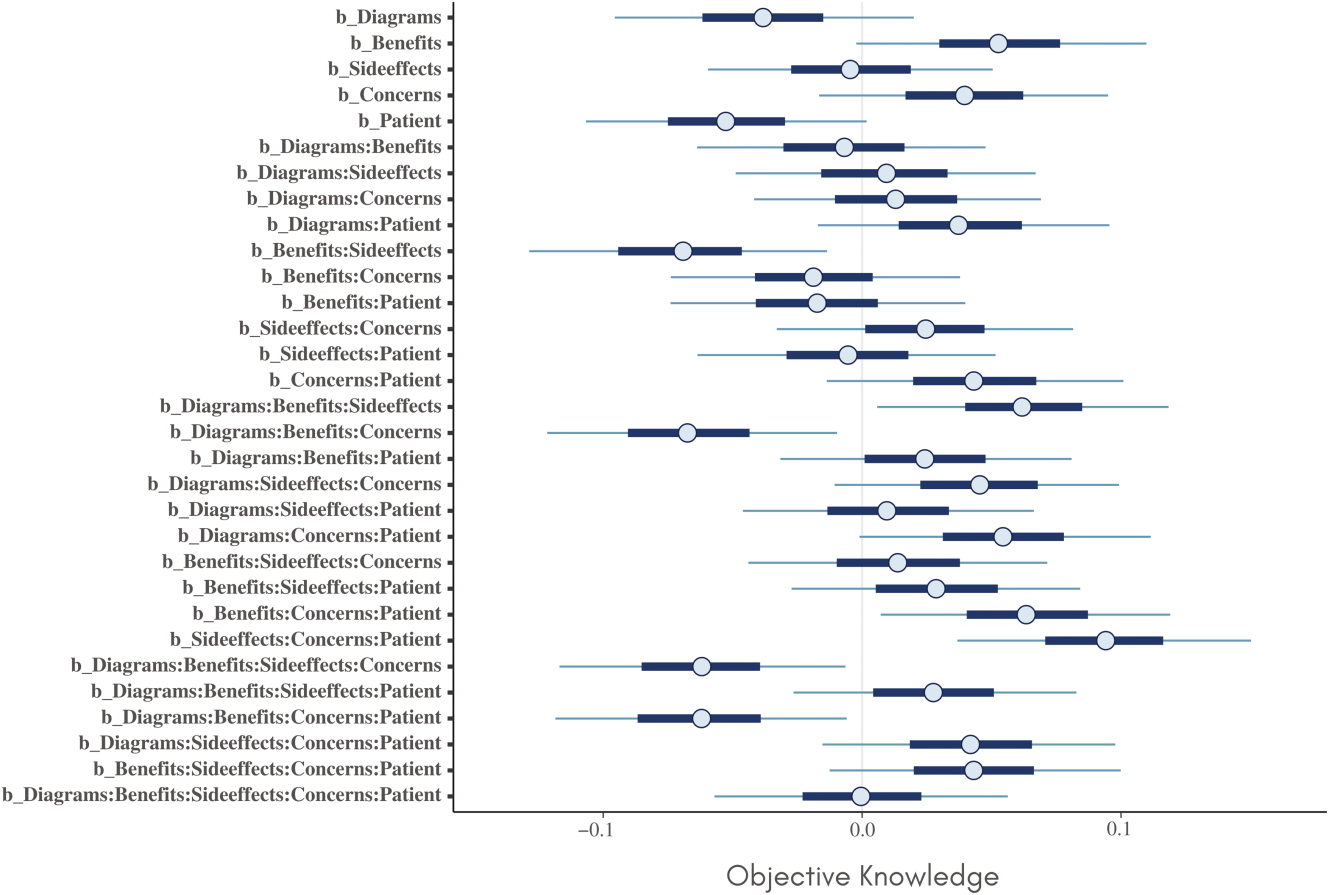
Credible intervals for the estimated main and interaction effects on objective knowledge.

**Figure 3:**
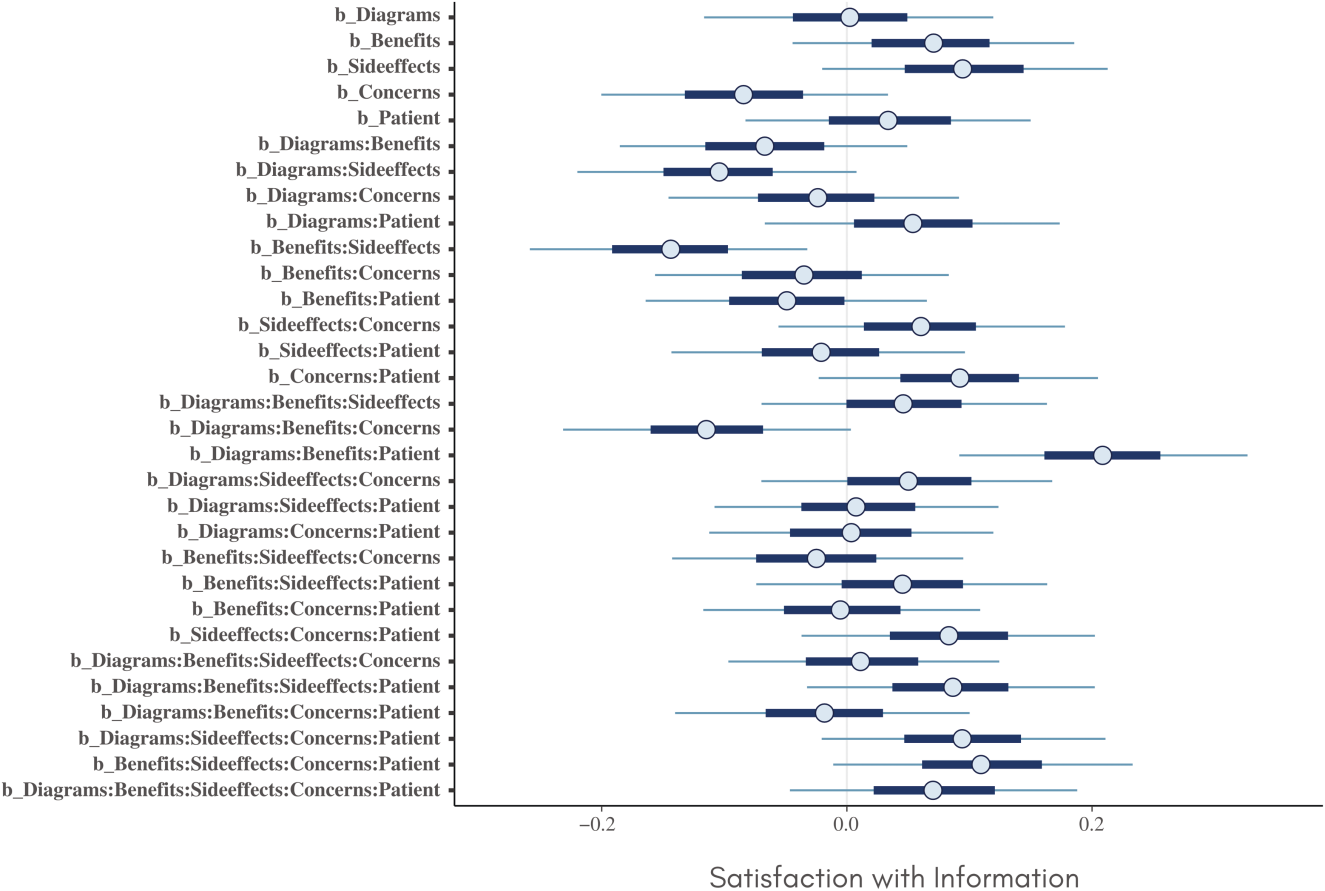
Credible intervals for the estimated main and interaction effects on satisfaction with information.

Credible intervals for the estimated main and interaction effects on each of the three outcomes are provided in Figs. 1–3. Of particular note is a main effect for the *patient* factor on *beliefs* (which was also reported by Green *et al*. [13]). There are also multiple interaction effects for which the credible interval does not include zero, suggesting that components are involved in complex synergistic and antagonistic effects on all three outcomes.

### Identifying an optimized leaflet

Table 2 provides expected values for all 32 alternative versions of the information leaflet, in order of magnitude. The optimized leaflet—i.e. the one with the largest expected value—is highlighted; in this leaflet, side-effects and patient were set to their higher levels, while all other factors were set to their lower levels.

**Table 2:** Expected values for all 32 versions of the information leaflet using multiple valued outcomes

Factors set to higher level	*D*	*B*	*SE*	*C*	*P*	Expected value
*SE, P*						0.668
*D, B, P*						0.654
*D, SE, C, P*						0.609
*D, B, SE, C, P*						0.607
*SE*						0.574
*SE, C*						0.574
*D, B, C, P*						0.547
*C, P*						0.540
*P*						0.537
*D, C, P*						0.537
*D, B, C*						0.531
*D, B, SE*						0.528
*B, SE*						0.519
*D, B, SE, P*						0.514
*B*						0.512
*B, C, P*						0.501
*B, SE, C, P*						0.497
*C*						0.495
*B, SE, C*						0.495
*D, SE, C*						0.494
*B, P*						0.493
*D, P*						0.484
*D, B*						0.484
*D*						0.483
*B, C*						0.482
*SE, C, P*						0.446
*D, SE*						0.425
*B, SE, P*						0.425
*D, SE, P*						0.403
*D, C*						0.393
*D, B, SE, C*						0.340
None						0.330

*Note. D = Diagrams; B = Benefits; SE = Side-effects; C = Concerns; P = Patient*. Cells highlighted in green indicate factors set to their higher level. The cell highlighted in grey identifies the optimized leaflet, or the version with the largest expected value.

### Exploring robustness

When we upheld the spirit of our initial preferences, in which *medication beliefs* was the primary outcome, followed in relative importance by *objective knowledge* and then *satisfaction with information*, we observed consistency in the choice of optimized intervention (Table 3). In Step 1, when we systematically decreased the importance of *satisfaction with information*, first, and then *objective knowledge*, the choice remained the same (i.e. *side-effects* and *patient* set to their higher levels) across all levels of relative importance, even when both *satisfaction with information* and *objective knowledge* had minimal importance. Similarly, in Step 2, when we systematically increased the importance of *objective knowledge*, first, and then *satisfaction with information*, the choice remained the same (*side-effects* and *patient* set to their higher levels) across all levels of relative importance, even when all three outcomes had equal importance (or points = 100 for all 3).

**Table 3:** Systematic variations in relative importance for three outcomes

Points			Factors set to their higher level in the selected optimized leaflet
*Step 1. Systematically reducing the importance of secondary outcomes*.			
*MB*	*OK*	*SI*	
100	50	25	*SE, P*
100	50	15	*SE, P*
100	50	5	*SE, P*
100	40	5	*SE, P*
100	30	5	*SE, P*
100	20	5	*SE, P*
100	10	5	*SE, P*
100	5	5	*SE, P*
*Step 2. Systematically increasing the importance of secondary outcomes*.			
*MB*	*OK*	*SI*	
100	50	25	*SE, P*
100	60	25	*SE, P*
100	70	25	*SE, P*
100	80	25	*SE, P*
100	90	25	*SE, P*
100	100	25	*SE, P*
100	100	35	*SE, P*
100	100	45	*SE, P*
100	100	55	*SE, P*
100	100	65	*SE, P*
100	100	75	*SE, P*
100	100	85	*SE, P*
100	100	100	*SE, P*

*Note.* Outcomes*: MB = Medication Beliefs; OK = Objective Knowledge; SI = Satisfaction with Information.* Factors*: D = Diagrams; B = Benefits; SE = Side-effects; C = Concerns; P = Patient*.

This level of consistency was not observed in the selected scenarios that deviated more markedly from our initial preferences. Table 4 reports the decisions under these scenarios. In general, these results suggest a pattern in which the choice of optimized intervention differs when the *satisfaction with information* importance outcome is relatively more important than the *objective knowledge* outcome. Furthermore, these results highlight the similarity in performance for the top two contenders for the optimized leaflet, the version with *side-effects* and *patient* set to their higher levels and the version with *diagrams*, *benefits*, and *patient* set to their higher levels; we return to this in the discussion.

**Table 4:** Preference scenarios that deviate from our initial preferences

Scenario	Points for outcomes			Description	Decision
	*MB*	*OK*	*SI*		
Initial preferences	100	50	25		*SE, P*
Scenario 1	100	25	50	Scenario in which the relative importance of *objective knowledge* and *satisfaction with information* is reversed.	*D, B, P*
Scenario 2	100	0	0	Scenario in which *medication beliefs* is the single primary outcome.	*D, B, P*
Scenario 3	0	100	0	Scenario in which *objective knowledge* is the single primary outcome.	*B*
Scenario 4	0	0	100	Scenario in which *satisfaction with information* is the single primary outcome.	*D, B, P*
Scenario 5	100	100	0	Scenario in which two outcomes, *medication beliefs* and *objective knowledge*, are equally important.	*SE, P*
Scenario 6	100	0	100	Scenario in which two outcomes, *medication beliefs* and *satisfaction with information*, are equally important.	*D, B, P*

*Note.* Outcomes*: MB = Medication Beliefs; OK = Objective Knowledge; SI = Satisfaction with Information. D = Diagrams; B = Benefits; SE = Side-effects; C = Concerns; P = Patient*.

## Discussion

Using empirical data from a 2^5^ factorial trial [13] and the latest recommendations for decision-making in MOST [11], we identified an optimized information leaflet for women with breast cancer that included two of our five candidate intervention components set to their higher levels: information about side-effects of the medication and patient input (quotes and photos) from survivors explaining their motivations for taking the medication. To identify this optimized leaflet, we applied the newly recommended approach for optimization decision-making, DAIVE, which offers advantages relative to the previously recommended approach, CSA—especially in terms of the ready accommodation of multiple outcome variables. The identified optimized leaflet demonstrated the best-expected performance in terms of three-valued outcomes: *medication beliefs*, *objective knowledge*, and s*atisfaction with information*. These outcomes were valued as hypothesized antecedents of eventual adherence to AET for women with breast cancer.

When Green *et al*. [13] previously applied CSA to identify an optimized information leaflet, that leaflet version differed from the optimized leaflet we identified here. The previous selected leaflet included four of the five candidate components set to their higher levels (*D, B, C,* and *P*)—and notably, included the lower level of the side-effects component. Since CSA can only accommodate a single outcome, decision-making in Green *et al*. [13], was based on *medication beliefs* only, so the differences between these two identified leaflets are not necessarily surprising. Instead, the differences are likely due in part to varying patterns of component performance on the three outcomes, suggesting a different choice when three outcomes versus one outcome are considered. However, one of the alternative preference scenarios we considered (Scenario 2, in which *medication beliefs* was the single primary outcome), suggests that these differences may also reflect other points of contrast between CSA and DAIVE. With the same outcome, CSA (as applied in Green *et al*. [13]) and DAIVE (in Scenario 2) identified different optimized interventions (diagrams, benefits, concerns and patient, and diagrams, benefits and patient, respectively). This is also not surprising; simulated testing of the two approaches across varied trials with one outcome variable, found that CSA and the methodology DAIVE uses (a posterior expected value approach) frequently but not always arrived at the same optimized interventions [11]. In this case, differences may reflect the judgment calls that went into interpreting higher-order interaction effects [13], as is common in applications of CSA.

To examine whether our choice of optimized intervention was susceptible to differences in the weights that assigned relative importance to the three outcomes, we systematically varied the points for the three outcomes (“points” referring to those used in a swing weighting exercise [36]). In general, our choice of optimized intervention was robust to systematic variations in weights for the three outcome variables that upheld the spirit of our initial preferences, supporting the idea that an optimized leaflet with side-effects and patient set to their higher levels is appropriate given our initial preferences. In contrast, when we considered scenarios that deviated from those we set initially—e.g. by giving *satisfaction with information* more importance than *objective knowledge*—there were differences in the choice of optimized intervention. This emphasizes the importance of making initial determinations about the relative importance of outcomes; for investigators using MOST, it may even be best to outline such determinations during the Preparation phase, e.g. as a conceptual model is being built.

Across the full range of relative importance we considered for the three outcomes, there were two top contenders for the optimized leaflet: the version with side-effects and patient set to their higher levels and the version with diagrams, benefits, and patient set to their higher levels. Even under our precise initial preferences for the three outcomes, the expected values of these two leaflet versions were close in magnitude, especially relative to the other possible combinations of component levels. This finding is not unique to our use of DAIVE for decision-making; in Green *et al*.’s previous application of CSA, alternative versions of the information leaflets also performed similarly [13]. We respond to this finding with the following observations:

First, the similarity in performance is unsurprising given the context, as components distinguished elements of an information leaflet. Furthermore, in two cases, components were operationalized as factors with higher versus lower levels (e.g. *side-effects*), meaning that the main effect estimated was for the higher level of the factor relative to the lower level, not for the presence versus absence of that component, potentially leading to smaller differences in performance across leaflet versions. Still, results suggest particular support for the patient component; the factor for *patient* had a significant main effect on *medication beliefs* and is included in both contenders for the optimized leaflet (side-effects, patient and diagrams, benefits, patient) at its higher level. The question appears to be which synergies with *patient* should be chosen, and there appears to be an either/or, perhaps due to small antagonisms between *side-effects* and either *diagrams* or *benefits*: the choice is either synergies of *patient* with *side-effects* or synergies of *patient* with *diagrams* and *benefits*, but not of *patient* with *side-effects, diagrams, and benefits*. Such antagonisms may have implications for health communication strategies, suggesting that certain types of messaging, effecting change via certain mechanisms, work better together (or worse) than others. In future work, factorial mediation analysis [37] could help to elucidate the mechanisms underlying communication strategies’ effects (e.g. reflecting different theoretical constructs, such as gain versus loss framing [38]).

Second, our objective in this trial was not to prove that one version of the information leaflet is definitively better than another version. In general, that is not the objective in any prototypical ORCT; “optimized” does not necessarily mean “best.” In other ORCTs similar performance for two alternative intervention versions may be further contextualized with cost data; if two interventions that perform similarly in terms of expected value have sufficiently different costs, a choice between them may be clear—and the choice may favor the version with slightly lower expected value, given large cost savings. In such a case, the ORCT provides very useful information that an alternative intervention version produces comparable expected benefits at a lower cost. We refer interested readers to Strayhorn *et al*. [14] for an example and further discussion of this. In the present trial to optimize the information leaflet, we did not consider monetary costs, primarily given the granularity in components and almost identical costs across leaflet versions. However, there may still be reason to think carefully about the benefits of smaller versus larger intervention packages, given very similar performance. Based on constraints of attention and time, participants could be more likely to engage in all components of a leaflet that contains fewer components set to their higher levels (i.e. side-effects and patient input) than they are to engage in all components of a leaflet that contains more components set to their higher levels (i.e. diagrams, benefits, and patient input set to their higher levels). This could be important, given the extent to which leaflet benefits seem to depend on synergies with the patient input component. In any case, the next step for this work is to test a selected optimized leaflet further, not to proceed directly to implementation [27]. In a subsequent, larger-scale ORCT, an optimized information leaflet will serve as one of four candidate intervention components (e.g. alongside text messages, a therapy program, and a side-effect self-management website). For this ORCT, the primary outcome of interest will be adherence to AET, and the target of the information leaflet will be medication beliefs [5]; enabling the hypotheses linking the antecedents we consider here (e.g. medication beliefs) to adherence to be tested.

Third, these results highlight the idea that interpreting ORCT results to mean that certain components are “inactive” may be unhelpful. DAIVE’s standard output (Table 3) offers a reminder that, when activity is defined in terms of desirable main effects and/or synergies in interaction effects, the differences between an intervention version that contains a particular component and another version that does not may be small. Therefore, to interpret such a difference as a signal of “inactivity” may be incorrect.

### Limitations and future directions

The sample of healthy women could limit generalizability of findings to women with breast cancer, who may have had differing beliefs about the medication, more prior knowledge about AET, and differing satisfaction with information. However, the baseline beliefs differential score was not significantly different between those who did (*n* = 79) and did not report a diagnosis of breast cancer, as discussed elsewhere [13]. Furthermore, the leaflet will now undergo further testing in a larger ORCT in women with breast cancer [27]. Additionally, we did not assess participant’s baseline AET knowledge, which could have impacted our findings.

It is currently usual practice in MOST for the preferences for multiple outcomes to be decided by the intervention scientists leading an ORCT [14]; our initial preferences for the three outcomes were defined by two investigators (S.M.C.G. and S.G.S.). It is plausible that, if other interested parties (e.g. clinicians and women with breast cancer) had been included in assigning relative importance to outcomes, we could have come to a different choice for the optimized intervention. Future work should explore the best strategies for incorporating wider perspectives in the determination of preferences in intervention optimization, likely drawing on a large literature in stakeholder engagement [39, 40], and preference elicitation [41, 42].

Furthermore, we relied on the number of candidate components in a given leaflet to indicate the efficiency (fewer components) versus complexity (more components) of that leaflet. While this strategy may be appropriate given the inevitable constraints on patients’ time and attention, it may also not fully capture patients’ subjective experiences. For example, it could be that candidate components (say, benefits versus concerns) produce different degrees of perceived burden. If we had quantified the burden associated with different leaflet versions more directly, we might have been able to strategically balance expected value with burden. It could be that this would have further affirmed the choice of a leaflet with fewer components (side-effects and patient input, versus diagrams, benefits, and patient input) set to their higher levels; of course, this could also have informed selection of a different optimized leaflet, especially since the top two contenders for the optimized leaflet performed so similarly in terms of expected value. In general, investigators using MOST may benefit from devoting some thought in the Preparation phase to how they might anticipate proceeding with selection of an optimized intervention in the event that two interventions perform similarly. One option would be to quantify uncertainty more explicitly than we did here; future work could expand on these possibilities.

### Conclusions

Based on our preferred combination of *medication beliefs*, *objective knowledge*, and *satisfaction with information,* the optimized leaflet contained the higher levels of the side-effects and patient input components. Consistent with the decision-making reported by Green *et al*. [13], the patient input component showed the strongest evidence for activity, with further evidence of synergies with other components. Using DAIVE to consider multiple valued outcomes highlighted potential in the side-effects component, which may have been otherwise overlooked.

## Supplementary Material

ibae029_suppl_Supplementary_Appendix_S1

ibae029_suppl_Supplementary_Appendix_S2

## Data Availability

De-identified data associated with this paper are available from https://doi.org/10.5518/1467

## Welfare of Animals

This article does not contain any studies with animals performed by any of the authors.

## Transparency Statement

*Study Registration:* The study was not formally registered. *Analytic Plan Pre-registration:* The analysis plan was not formally pre-registered. *Analytic Code Availability:* Analytic code used to conduct the analyses presented in the current study is available in the appendices. *Materials Availability:* Materials used to conduct the study are available at https://doi.org/10.17605/OSF.IO/AG7YK.
