# Supplementary material for: Decision-making in the multiphase optimization strategy: Applying decision analysis for intervention value efficiency to optimize an information leaflet to promote key antecedents of medication adherence
Source: Transl Behav Med. 2024 May 25;14(8):461–71. doi: 10.1093/tbm/ibae029 (PMC11282575; doi:10.1093/tbm/ibae029)
Supplement: ibae029_suppl_Supplementary_Appendix_S1 [file ibae029_suppl_supplementary_appendix_s1.docx]

| Component | Higher level (on or enhanced) | Lower level (off or basic) |
| --- | --- | --- |
| Constant component | N/A. The constant component was provided to all participants. It contained the title, logo, types of hormone therapy, text description of how AET works, and information about how to take AET, how to get more tablets, what to do if missed a dose, taking AET with other drugs, and information if wanting to stop AET. | |
| Diagrams | ON: Three diagrams of how AET works. One explaining how oestrogen helps breast cancer to grow, one explaining the mechanism of tamoxifen and one explaining the mechanism or aromatase inhibitors (different types of AET). | OFF: Text only explaining how oestrogen is linked to breast cancer, how tamoxifen works, and how aromatase inhibitors work. |
| Benefits | ENHANCED: One page describing the benefits of AET in terms of reduced recurrence and mortality. Two icon arrays are included to demonstrate how AET can reduce mortality. | BASIC: Two bullet points stating that AET can reduce risk of recurrence and mortality. |
| Side-effects | ENHANCED: Detailed side-effects table with information about the prevalence of each type of side-effect for each type of AET. Prevalence statistics included positively framed statements (e.g., 99% of women will not experience this side-effect). An additional paragraph titled “are all my symptoms because of the medication I am taking” described the nocebo effect, and suggested some of these symptoms may be experienced without taking AET, e.g. during the menopause. | BASIC: Prevalence table which stated which side-effects might be present when taking each type of AET, but did not go into any detail about the prevalence of the side-effects. |
| Concerns | ON: Four common concerns with answers; worrying about experiencing a lot of side-effects, worrying about not being able to cope with side-effects, actually experiencing side-effects, and worry about getting another type of cancer. | OFF: Absent- no concerns or answers included. |
| Patient | ON: On the title page: 4 pictures of women who provided quotes for the leaflet, and a statement to say the leaflet has been designed with input from breast cancer survivors.  Distributed throughout, there are four quotes from women with AET about their motivations to take the AET. | OFF: Absent- no quotes or pictures includes. |

**Appendix 1: Descriptions of candidate intervention components and factor levels**
