# Supplementary material for: Decision-making in the multiphase optimization strategy: Applying decision analysis for intervention value efficiency to optimize an information leaflet to promote key antecedents of medication adherence
Source: Transl Behav Med. 2024 May 25;14(8):461–71. doi: 10.1093/tbm/ibae029 (PMC11282575; doi:10.1093/tbm/ibae029)
Supplement: ibae029_suppl_Supplementary_Appendix_S2 [file ibae029_suppl_supplementary_appendix_s2.docx]

**Appendix 2- R code for analyses**

**##Analytic Code**

**###BMQ = Medication beliefs**

**###SIMS = Satisfaction with information**

**###Know = Objective knowledge**

**#Bayesian Factorial ANOVA**

BMQmodel = brm(BMQ2Diff ~ BMQ1Diff + (Diagrams + Benefits + Sideeffects + Concerns + Patient)^5, data = ldata, prior=prior("normal(0,5"), chains=4, backend = "cmdstanr")

summary(BMQmodel)

SIMSmodel = brm(SIMSTotalNew ~ (Diagrams + Benefits + Sideeffects + Concerns + Patient)^5, data = ldata, prior=prior("normal(0,5"), chains=4, backend = "cmdstanr")

summary(SIMSmodel)

Knowmodel = brm(KnowTotal ~ (Diagrams + Benefits + Sideeffects + Concerns + Patient)^5, data = ldata, prior=prior("normal(0,5"), chains=4, backend = "cmdstanr")

summary(Knowmodel)

**#Creating effect codes**

#Codes for BMQ are different because we controlled for BMQ at baseline

codesBMQ = expand.grid(Intercept = 1,

BMQDiff1 = 0,

D = c(-1,1),

B = c(-1,1),

SE = c(-1,1),

C = c(-1,1),

P = c(-1,1))

setDT(codesBMQ)

codesBMQ[, DxB := D * B]

codesBMQ[, DxSE := D * SE]

codesBMQ[, DxC := D * C]

codesBMQ[, DxP := D * P]

codesBMQ[, BxSE := B * SE]

codesBMQ[, BxC := B * C]

codesBMQ[, BxP := B * P]

codesBMQ[, SExC := SE * C]

codesBMQ[, SExP := SE * P]

codesBMQ[, CxP := C * P]

codesBMQ[, DxBxSE := D * B * SE]

codesBMQ[, DxBxC := D * B * C]

codesBMQ[, DxBxP := D * B * P]

codesBMQ[, DxSExC := D * SE * C]

codesBMQ[, DxSExP := D * SE * P]

codesBMQ[, DxCxP := D * C * P]

codesBMQ[, BxSExC := B * SE * C]

codesBMQ[, BxSExP := B * SE * P]

codesBMQ[, BxCxP := B * C * P]

codesBMQ[, SExCxP := SE * C * P]

codesBMQ[, DxBxSExC := D * B * SE * C]

codesBMQ[, DxBxSExP := D * B * SE * P]

codesBMQ[, DxBxCxP := D * B * C * P]

codesBMQ[, DxSExCxP := D * SE * C * P]

codesBMQ[, BxSExCxP := B * SE * C * P]

codesBMQ[, DxBxSExCxP := D * B * SE * C * P]

setDT(codesBMQ)

**#Codes for SIMS and Know**

codes = expand.grid(Intercept = 1,

D = c(-1,1),

B = c(-1,1),

SE = c(-1,1),

C = c(-1,1),

P = c(-1,1))

setDT(codes)

codes[, DxB := D * B]

codes[, DxSE := D * SE]

codes[, DxC := D * C]

codes[, DxP := D * P]

codes[, BxSE := B * SE]

codes[, BxC := B * C]

codes[, BxP := B * P]

codes[, SExC := SE * C]

codes[, SExP := SE * P]

codes[, CxP := C * P]

codes[, DxBxSE := D * B * SE]

codes[, DxBxC := D * B * C]

codes[, DxBxP := D * B * P]

codes[, DxSExC := D * SE * C]

codes[, DxSExP := D * SE * P]

codes[, DxCxP := D * C * P]

codes[, BxSExC := B * SE * C]

codes[, BxSExP := B * SE * P]

codes[, BxCxP := B * C * P]

codes[, SExCxP := SE * C * P]

codes[, DxBxSExC := D * B * SE * C]

codes[, DxBxSExP := D * B * SE * P]

codes[, DxBxCxP := D * B * C * P]

codes[, DxSExCxP := D * SE * C * P]

codes[, BxSExCxP := B * SE * C * P]

codes[, DxBxSExCxP := D * B * SE * C * P]

setDT(codes)

**#Posterior densities**

**#BMQ outcome**

BMQ_post = spread_draws(BMQmodel, `b_.*`, regex = TRUE)

mcmc_intervals(BMQ_post[,6:36])

BMQ_post_DT = setDT(BMQ_post)

reslist <- vector("list", length = 4000)

for(i in 1:4000){

reslist[[i]] <- as.matrix(codesBMQ) %*% t(as.matrix(BMQ_post_DT[.draw == i,4:36]))

}

BMQ_outcomes = data.frame(D = rep(c(codesBMQ$D), 4000),

B = rep(c(codesBMQ$B), 4000),

SE = rep(c(codesBMQ$SE), 4000),

C = rep(c(codesBMQ$C), 4000),

P = rep(c(codesBMQ$P), 4000),

BMQ = unlist(reslist))

setDT(BMQ_outcomes)

#mcmc_areas_ridges(BMQ_outcomes, pars = "BMQ")

#cred1 = mcmc_intervals(BMQ_post[,6:36])

**#SIMS outcome**

SIMS_post = spread_draws(SIMSmodel, `b_.*`, regex = TRUE)

cred2 = mcmc_intervals(SIMS_post[,5:35])

SIMS_post_DT = setDT(SIMS_post)

reslist_SIMS <- vector("list", length = 4000)

for(i in 1:4000){

reslist_SIMS[[i]] <- as.matrix(codes) %*% t(as.matrix(SIMS_post_DT[.draw == i,4:35]))

}

SIMS_outcomes = data.frame(D = rep(c(codes$D), 4000),

B = rep(c(codes$B), 4000),

SE = rep(c(codes$SE), 4000),

C = rep(c(codes$C), 4000),

P = rep(c(codes$P), 4000),

SIMS = unlist(reslist_SIMS))

setDT(SIMS_outcomes)

#mcmc_areas_ridges(SIMS_outcomes, pars = "SIMS")

**#KnowTotal outcome**

Know_post = spread_draws(Knowmodel, `b_.*`, regex = TRUE)

cred3 = mcmc_intervals(Know_post[,5:35])

Know_post_DT = setDT(Know_post)

reslist_Know <- vector("list", length = 4000)

for(i in 1:4000){

reslist_Know[[i]] <- as.matrix(codes) %*% t(as.matrix(Know_post_DT[.draw == i,4:35]))

}

Know_outcomes = data.frame(D = rep(c(codes$D), 4000),

B = rep(c(codes$B), 4000),

SE = rep(c(codes$SE), 4000),

C = rep(c(codes$C), 4000),

P = rep(c(codes$P), 4000),

Know = unlist(reslist_Know))

setDT(Know_outcomes)

#mcmc_areas_ridges(Know_outcomes, pars = "Know")

**#Full data.frame**

Outcome_data = cbind(BMQ_outcomes, SIMS_outcomes$SIMS, Know_outcomes$Know)

Outcome_data = rename(Outcome_data, SIMS = V2)

Outcome_data = rename(Outcome_data, Know = V3)

#mcmc_areas_ridges(Outcome_data, pars = c("BMQ", "SIMS", "Know"))

**#Scaling**

minBMQ = min(Outcome_data$BMQ)

maxBMQ = max(Outcome_data$BMQ)

minSIMS = min(Outcome_data$SIMS)

maxSIMS = max(Outcome_data$SIMS)

minKNOW = min(Outcome_data$Know)

maxKNOW = max(Outcome_data$Know)

Outcome_data$BMQscale = (Outcome_data$BMQ - minBMQ)/(maxBMQ-minBMQ)

Outcome_data$SIMSscale = (Outcome_data$SIMS - minSIMS)/(maxSIMS-minSIMS)

Outcome_data$KNOWscale = (Outcome_data$Know - minKNOW)/(maxKNOW-minKNOW)

**#Visualizing outcomes**

#mcmc_areas_ridges(Outcome_data, pars = c("BMQscale", "SIMSscale", "KNOWscale"))

names = c("NULL", "A", "B", "AB", "C", "AC", "BC", "ABC", "D", "AD", "BD", "ABD", "CD", "ACD", "BCD", "ABCD", "E", "AE", "BE", "ABE", "CE", "ACE", "BCE", "ABCE", "DE", "ADE", "BDE", "ABDE", "CDE", "ACDE", "BCDE", "ABCDE")

Outcome_data$names = rep(names, 4000)

**#Combining outcomes**

Value_summary = codes[,2:6]

Value_summary$BMQ = NA

Value_summary$SIMS = NA

Value_summary$Know = NA

for (i in 1:32) {

Sub = match_df(Outcome_data, Value_summary[i,1:5])

Value_summary$BMQ[i] = mean(Sub$BMQscale)

Value_summary$SIMS[i] = mean(Sub$SIMSscale)

Value_summary$Know[i] = mean(Sub$KNOWscale)

}

Value_summary$names = names

**#Initial outcome weights**

weight1 = 100/175

weight2 = 50/175

weight3 = 25/175

weightedsum = as.numeric(cbind(Value_summary$BMQ, Value_summary$SIMS, Value_summary$Know)%*%c(weight1, weight2, weight3))

Value_summary$EV = weightedsum
